# Are amblyopia therapy outcomes related to socioeconomic situation for children in Greater Manchester?

**DOI:** 10.1038/s41433-026-04280-z

**Published:** 2026-03-13

**Authors:** Laura England, Catherine Fullwood, Jignasa Mehta, Kerry Hanna, Anna O’Connor

**Affiliations:** 1 Manchester Royal Eye Hospital, Manchester University NHS Foundation Trust, Manchester, UK; 2 University of Liverpool, Liverpool, UK; 3 Manchester University NHS Foundation Trust, Manchester, UK; 4 University of Manchester, Manchester, UK

**Keywords:** Vision disorders, Paediatrics, Public health, Risk factors

## Abstract

**Background:**

Limited evidence exists around health inequalities in amblyopia therapy. This cohort study explores amblyopia therapy outcomes and socioeconomic scores by postcode, across two boroughs within Greater Manchester.

**Methods:**

All available orthoptic records for school vision screening referrals from the academic year 2017–2018 were reviewed by one Research Orthoptist, to identify children diagnosed with unilateral amblyopia. Clinical data were extracted and the proportion amblyopia deficit corrected and appointment attendance rates over 1 year of therapy were calculated. Home postcodes were used to identify socioeconomic situation for each patient, by Index of Multiple Deprivation (IMD) 2019 and Townsend Deprivation Index 2011.

**Results:**

From 730 school screening referrals, 512 orthoptic records were accessible and 42 cases of unilateral amblyopia were identified. The median proportion amblyopia deficit corrected in 1 year was 51.0% (IQR 22.6–72.9). The median attendance rate was 87.5% (IQR 67.9–100.0); 47.6% of patients attended every appointment. No statistically significant relationships were found between socioeconomic score and the proportion amblyopia deficit corrected in a year (IMD: unstandardised beta coefficient 1.782, 95% CI −1.877 to 5.441, *p* = 0.331) or orthoptic clinic attendance rates (IMD: unstandardised beta coefficient −0.479, 95% CI −2.492 to 1.534, *p* = 0.633). A positive relationship was found between attendance rate and proportion amblyopia deficit corrected (unstandardised beta coefficient 0.743, 95% CI 0.213 to 1.274, *p* = 0.007).

**Conclusion:**

In two Greater Manchester community orthoptic services, amblyopia therapy outcome and clinic attendance rate were not related to individual socioeconomic scores by postcode. A positive relationship between orthoptic clinic attendance rate and amblyopia therapy outcome was found.

## Introduction

Amblyopia is a developmental disorder of vision, not involving structural anomalies or ocular disease, which affects ~2% of children [1]. It is a target condition to identify in UK school vision screening programmes [2].

Amblyopia therapy is effective [3, 4], but reported success rates vary from 19 to 93% [5]. There is, however, no universally agreed method of defining amblyopia therapy success. Stewart et al. [6] devised a ‘proportion of deficit corrected’ calculation, which considers: the amblyopic vision at the start and end of therapy; vision of the fellow eye; and visual development that may naturally occur during the therapy period.

One factor that affects the success of amblyopia therapy is how closely the therapy undertaken matches what is prescribed [5, 7, 8]. When measured objectively, concordance with occlusion therapy is often around 50% [9]. Factors reported to improve concordance include: recent commencement of occlusion therapy [10, 11]; increased parental understanding [12], confidence [13] and involvement in therapy [14]; and advantaged socioeconomic situation (SES) [9].

SES describes the relative advantage or disadvantage that an individual or group experiences in accessing and controlling economic, material and social resources or opportunities [15]. In England, SES is often measured by the English Index of Multiple Deprivation (IMD) [16], which consists of seven domains that contribute to an overall score: income; employment; health; education; barriers to housing and services; living environment; and crime. The Townsend Deprivation Index [17] is another socioeconomic scale, used by UK health authorities due to its correlation with ill health [18]. It comprises four domains: unemployment; household overcrowding; non-car ownership; and non-home ownership.

A relationship between socioeconomic disadvantage and increased amblyopia prevalence is suggested by O’Colmain et al. [19], who report on 1170 children in Tayside, Scotland, following pre-school vision screening. Children were 1.4 times more likely to pass the assessment if they were advantaged (by the Scottish IMD score [SIMD]) and three times more likely to fail if they were from homes needing more support from services (measured by the Health Plan Indicator [HPI], a support category assigned to each child by their Health Visitor). A recent literature review [20] suggested that this relationship could be linked to risk factors for amblyopia that are also more prevalent in disadvantaged individuals, such as hypermetropia [21, 22] and maternal smoking in pregnancy [23, 24].

Three studies were found that explored a relationship between SES and amblyopia therapy outcomes during childhood [25–27]. A follow-on from the Tayside study [19] included 430 children who received orthoptic therapy after screening [25]. Children from families requiring more support by HPI category were more likely to have worse vision (odds ratio 5.37) and binocularity (odds ratio 3.41) outcomes than their advantaged peers, even when they had good attendance at orthoptic appointments. The SIMD score, however, did not affect the overall vision outcome when attendance was good. An American study [26] of 280 children found that those from families receiving Medicaid (the American public health insurance program for people with low income) showed worse final visual acuities in the amblyopic eye (58% of the non-Medicaid group achieved at least 6/9, compared to 27% of the Medicaid group, no confidence intervals reported). However, a different American study [27] of 73 children found no difference in visual outcome between Medicaid and private health insurance groups. In this study, patients had to attend at least twice to be included, and they were almost three times more likely to be lost to follow-up if they were from the Medicaid group, so the visual acuity analysis is potentially biased.

As suggested above, appointment attendance rate is one factor that may explain the suggested relationship between socioeconomic disadvantage and reduced therapy outcomes. In the Tayside study [25], disadvantaged children by SIMD score or HPI category were more likely (twice and almost four times as likely, respectively) to have poor attendance at hospital appointments. Poor attendance increased the odds of having residual amblyopia (odds ratio 6.42) and poor/no binocular vision at discharge (49% more likely). Children receiving Medicaid in the larger American cohort [26], also missed more appointments, although this was not quantified.

### Rationale

The studies described above provide some limited evidence of health inequalities for amblyopia therapy. None of them were conducted in England, demonstrating the need for further work. Greater Manchester is a highly populated area with a diverse range of socioeconomic scores [28], therefore it provides a suitable geographical region to investigate further whether amblyopia therapy outcomes are affected by SES.

### Study objective

To investigate whether a relationship between SES and amblyopia therapy outcome exists in Greater Manchester.

## Materials and methods

### Design

A retrospective cohort study was conducted across two boroughs within Greater Manchester: Manchester and Trafford. The boroughs were selected as the most and least disadvantaged overall, defined by the IMD 2019, to increase the range of participant IMD scores.

### Sample size

A pragmatic sample size of at least 40 eligible patient records (at least 15 from each borough) was selected, to be both feasible within the study timeline and to provide a robust number for analyses. Considering the primary analysis method, a univariate linear regression model, 40 participants would allow the detection of a small effect size of 0.2 with an alpha of 0.05 and 80% power.

### Inclusion criteria

- School-entry vision screening referral accepted by Manchester or Trafford orthoptic services from the academic year 2017–2018. This criterion identifies amblyopes of a similar age, to reduce potential bias resulting from a reduction in amblyopia therapy success with increasing age during childhood [5, 8, 29].
- Diagnosis of unilateral amblyopia, defined as an interocular VA difference of at least 0.2 logMAR, after refractive correction if required. Slightly smaller interocular differences were permitted if, for example, the clinician had commenced amblyopia therapy or diagnosed unilateral amblyopia: the Research Orthoptist collecting data used professional judgement to define inclusion. This criterion identifies those children eligible for occlusion therapy.

### Exclusion criteria

- Incomplete records for essential data collection.
- Records not available.
- Ocular pathology, to exclude other causes for reduced VA.
- Transferred to other orthoptic services during the 1-year period.
- 1-year period finishes after February 2020, to avoid effects of the COVID-19 pandemic.

### Procedures

A Research Orthoptist accessed the 2017–2018 orthoptic screening referral databases and recorded all available NHS numbers for referrals. If no NHS number was found, the patient was excluded at this point. Each available NHS number was checked on the National Data Opt-Out service; if any patient had opted out of their data being included in research (3%), their notes were not accessed. Available orthoptic clinic records were then accessed, at Manchester and Trafford community orthoptic clinics, to identify eligible school vision screening referrals from 2017 to 2018.

Between December 2022 and May 2023, the following details were extracted from eligible orthoptic records **(data in bold were essential for analysis): patient’s home postcode at amblyopia diagnosis or in 2017–2019;** the date, VA and test used at school and first clinic appointment; diagnosis at the first clinic appointment; first refraction and fundus check details; the **date, VA**, test used (to enable conversion of VA levels so that all scores were comparable), cover test, binocularity results and **full orthoptic diagnosis at amblyopia diagnosis**; **date of the orthoptic appointment closest to 1 year after amblyopia diagnosis** (up to 14 months afterwards), **1 year VA**, test used, cover test, binocularity results and orthoptic diagnosis at this point; orthoptic therapy received and the Research Orthoptist’s interpretation of concordance (none/1–30%/31–60%/61–90%/91% and over, excluding non-attendance) over the course of the year; reason for discharge/cessation of follow-up if this occurred during the year; **the number of booked and attended appointments related to orthoptic therapy in the year**; VA at all visits in the first year and the corresponding dates.

If a child’s carer stopped bringing the child to orthoptic appointments at any point during the ‘1 year’ period, without any knowledge that they were attending another service, their ‘1 year’ appointment was defined as the last one they were brought to, even if it were the appointment at which amblyopia was diagnosed. If orthoptic diagnoses were not recorded in the case notes where they were required for the study, the Research Orthoptist could make diagnoses from the reports, for data collection purposes.

### Data analysis

The primary outcome measure was proportion amblyopia deficit corrected within the first year of amblyopia diagnosis, which appropriately describes the outcome of amblyopia therapy as explained by Stewart et al. [6]. It was calculated for each participant, based upon the following formula:

$$\frac{({{{\rm{baseline}}}}\; {{{\rm{amblyopic}}}}\; {{{\rm{visual}}}}\; {{{\rm{acuity}}}} \; {{{\rm{\hbox{ - }}}}} \; {{{\rm{amblyopic}}}}\; {{{\rm{visual}}}}\; {{{\rm{acuity}}}}\; {{{\rm{at}}}} \, 1 \, {{{\rm{year}}}})}{({{{\rm{baseline}}}}\; {{{\rm{amblyopic}}}}\; {{{\rm{visual}}}}\; {{{\rm{acuity}}}} \; {{{\rm{\hbox{ - }}}}} \; {{{\rm{fellow}}}}\; {{{\rm{eye}}}}\; {{{\rm{visual}}}}\; {{{\rm{acuity}}}}\; {{{\rm{at}}}} \, 1 \, {{{\rm{year}}}})}$$

The secondary outcome measure was percentage attendance rate at orthoptic clinic appointments. This was calculated for each participant as follows:

$$\frac{{{{\rm{Number}}}}\; {{{\rm{of}}}}\; {{{\rm{appointments}}}}\; {{{\rm{attended}}}} \, \times 100}{{{{\rm{Number}}}}\; {{{\rm{of}}}}\; {{{\rm{appointments}}}}\; {{{\rm{booked}}}}}$$

SES was measured for each participant by IMD 2019 and Townsend 2011 scores. Deciles (IMD) or quintiles (Townsend), according to reporting convention for each index, were available online [30, 31] using each participant’s home postcode at amblyopia diagnosis (and any subsequent home postcodes where applicable).

Descriptive statistics and appropriate graphics were used to understand the data and basic relationships, then data were imported into the IBM statistics package, SPSS version 28.0.1.1 (14) for detailed statistical analysis. No imputation was made for any missing data. Univariate linear regression models were used to explore the relationships first between the primary outcome and SES, then for the secondary outcome, followed by multivariable models adjusting for baseline. Sensitivity analyses repeated the regression models for the primary outcome with an identified outlier removed, and with only those participants who had at least 10 months’ follow-up.

### Ethical statement

This study was performed in accordance with The Declaration of Helsinki. Ethical approval was given by the Wales Research Ethics Committee 4 (reference 22/WA/0278).

## Results

### Sample identification

The screening service databases recorded 730 school vision screening referrals in the academic year 2017–2018. Of these, 512 (70.1%) relevant orthoptic records were available to view. Four-hundred and seventy (64.4%) were excluded (reasons detailed in Table 1), leaving 42 (5.8%) patient records eligible for inclusion in this study.

**Table 1:** Reasons why available records were excluded.

Reason for exclusion	Number of records (%)
Not unilateral amblyopia – VA satisfactory in both eyes	267 (56.8)
Patient non-attendance prevents minimum data collection	76 (16.2)
Not unilateral amblyopia – VA bilaterally reduced	66 (14.0)
Not unilateral amblyopia – VA difference too small	34 (7.2)
1 year period ends after Feb 2020 (effects of COVID pandemic)	10 (2.1)
Referred to/seen at other services	9 (1.9)
Unclear from data collection spreadsheet	4 (0.9)
Referred to ophthalmology	3 (0.6)
Insufficient data available for minimum data collection (reasons other than non-attendance)	1 (0.2)
**Total**	**470 (100)**

### Socioeconomic situation

**Fig. 1:**
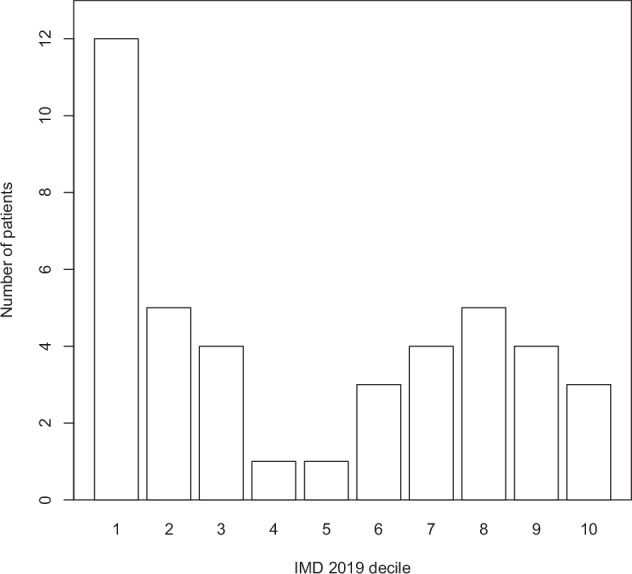
Distribution of all patients across IMD 2019 deciles. Each bar displays the number of patients (y-axis) from each IMD 2019 decile (x-axis), at the time of amblyopia diagnosis.

At the time of amblyopia diagnosis, the socioeconomic positions for the 42 eligible patients were distributed as shown in Fig. 1. It shows an uneven distribution across IMD deciles, with 28.6% of patients categorised as residing in the least advantaged decile, compared to only 2.4% in each of deciles 4 and 5.

### Clinical characteristics

Refractive amblyopia was the most prevalent type (54.8% anisometropic, 11.9% meridional, 14.3% anisometropic/meridional), followed by mixed (11.9%), then strabismic (2.4%). There was no identifiable cause for amblyopia in two patients (4.8%). Of the 42 patients included, 41 (97.6%) received glasses in the first year of therapy and 36 (85.7%) received patching therapy; none of the patients received atropine occlusion. Over three-quarters (76.2%) of parents reported more than 60% adherence with amblyopia therapy and over a third (35.7%) reported more than 90% adherence.

### Attendance at orthoptic appointments

The overall median (IQR) attendance rate was 87.5% (67.9–100.0), although just under half of patients (47.6%) attended every appointment. Four missed appointments were cancelled by parent/guardians and 31 were unattended without contact from the family.

The median (IQR) period of follow-up data was 11 (8–12) months. Twenty-seven patients (64.3%) had at least 10 months of follow-up data. For those with a shorter eligible follow-up, the most common reason for this (53.3% of patients) was children not being brought to their appointments. There was no clear link between SES and a longer duration of follow-up data.

The attendance rate was slightly worse for those with less than 10 months follow-up data (median 75.0%, IQR 50.0–100.0 versus median 87.5%, IQR 77.5–100.0), although the interquartile ranges are wide and overlapping. The most common type of amblyopia was anisometropic in both groups.

### Visual acuity

The median (IQR) logMAR VA for this cohort was 0.36 (0.30–0.50) at baseline and 0.20 (0.14–0.39) at 1 year for amblyopic eyes. For fellow eyes, it was 0.11 (0.10–0.15) at baseline and 0.08 (0.03–0.10) at 1 year. The median (IQR) proportion amblyopia deficit corrected at 1 year was 51.0% (22.6–72.9).

### Relationship between socioeconomic situation and proportion amblyopia deficit corrected in a year

**Fig. 2:**
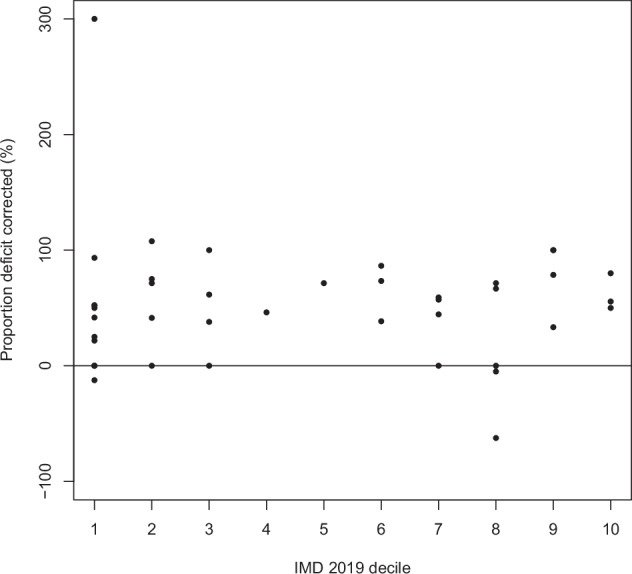
Proportion amblyopia defi cit corrected in a year against IMD 2019 decile. Points on the scatter plot indicate the IMD 2019 decile (x-axis) and the corresponding proportion amblyopia defi cit corrected (%) (y-axis) for individual patients.

**All data:** Fig. 2 is a scatter plot of the proportion amblyopia deficit corrected in a year against each patient’s overall IMD 2019 decile, for all 42 patients. A linear regression model found no relationship between the two variables, with an unstandardised beta coefficient of −0.325 (95% CI −5.545 to 4.895, *p* = 0.901). The findings did not differ for any individual IMD domains, or when using IMD ranks, IMD quintiles, or Townsend scores.

Figure 2 also suggests that one patient is a potential outlier, with 300% amblyopia improvement. On examination, this patient may not have had genuine unilateral amblyopia, therefore, they were excluded and the analyses involving amblyopia deficit correction repeated. No significant differences were found: for the overall IMD decile, the unstandardised beta coefficient was 1.782 (95% CI −1.877 to 5.441, *p* = 0.331).

### Patients with at least 10 months of follow-up data

Patients with at least 10 months of follow-up data had a greater proportion amblyopia deficit corrected (median 59.1%, IQR 48.1−76.8) compared to those with a shorter period (median 23.4%, IQR 0.00−40.52). Repeating the models for only those patients with at least 10 months of follow-up data found no change to the conclusions.

### Relationship between socioeconomic situation and attendance rate

**Fig. 3:**
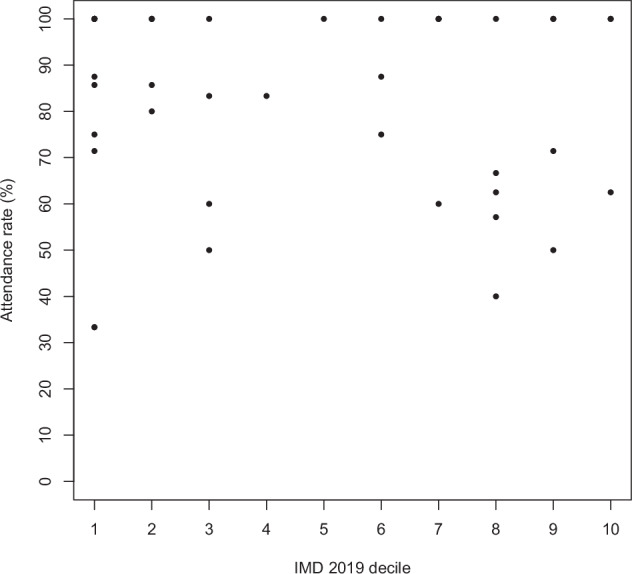
Patient attendance against IMD 2019 decile. Points on the scatter plot indicate the IMD 2019 decile (x-axis) and the corresponding attendance rate (%) (y-axis) for individual patients.

Figure 3 displays attendance rates for the full cohort; no clear relationship with IMD decile can be seen. A linear regression model also failed to identify a statistically significant relationship, with an unstandardised beta coefficient of −0.479 (95% CI −2.492 to 1.534, *p* = 0.633). These findings did not differ for any individual IMD domains, or when IMD ranks or Townsend scores were used.

### Relationship between attendance rate and proportion amblyopia deficit corrected in a year

**Fig. 4:**
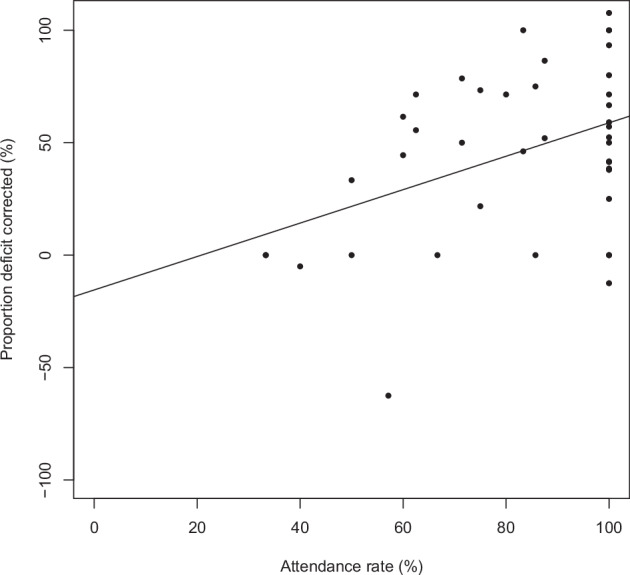
Proportion amblyopia defi cit corrected in a year against attendance rate. Points on the scatter plot indicate the attendance rate (%) (x-axis) and the proportion amblyopia deficit corrected in a year (%) (y-axis) for individual patients. The trend line shows a signifi cantly positive relationship (unstandardised beta coefficient 0.743, 95% CI 0.213 to 1.274, *p*= 0.007).

Figure 4 displays the proportion amblyopia deficit corrected in a year against attendance rate, excluding the outlier. The proportion deficit corrected was significantly positively related to attendance rate, with an unstandardised beta coefficient of 0.743 (95% CI 0.213 to 1.274, *p* = 0.007). This means that for every increase of 1% in attendance, the proportion amblyopia deficit corrected increases by 0.743%, as shown by the trend line on Fig. 4.

## Discussion

The primary aim of this study was to determine whether there is a relationship between SES and amblyopia therapy outcome using data from two Greater Manchester boroughs. No significant relationships were found using either IMD 2019 or Townsend 2011 scores.

Our results appear surprising, given the suggested relationship between disadvantage and reduced therapy outcomes reported by O’Colmain et al. [25] and Hudak and Magoon [26]. There were, however, methodological differences between their studies and ours. Hudak and Magoon [26] conducted their study in the USA, with a different healthcare system, almost 30 years ago so socioeconomic effects are likely to be different. O’Colmain et al. [25] separated ‘good’ and ‘poor’ attenders for analysis of visual outcomes and only related SES to visual outcome for those who attended well. Also, for this most relatable study [25], the relationship between disadvantage and reduced visual outcome was based upon children individually identified to require additional support (by HPI category). The same study [25], like ours, did not find a relationship using postcode-identified scores (the SIMD). This could suggest that socioeconomic scores identified by postcode are not personal enough, or not focussed on the relevant individual needs, to identify factors that influence amblyopia therapy outcome in the UK. The HPI score, which is not available in England (so was not available for our study), personally-identifies families with extra challenges in their lives, such as mental ill-health, poor social networks, housing difficulties, communication barriers, substance misuse, poverty, child protection issues, or chronically ill children. It seems feasible that if a family is identified as having one or more of these challenges, it could reduce their capacity to conduct occlusion therapy, whereas not all families within a particular postcode would have the same capacity for occlusion therapy.

The third study [27] to investigate a relationship between SES and amblyopia therapy outcomes during childhood, like ours found no relationship. However, they used raw amblyopic VA improvement, which does not consider baseline VA; those with worse starting VA tended to improve more so would have appeared to do better. Also, patients were only included in the VA analysis if they attended at least once after amblyopia diagnosis and the proportion of Medicaid patients who were excluded for this reason was significantly greater (28% vs 10%, *p* = 0.001) than patients with private insurance, which could have skewed their results. Other evidence also suggests a relationship between socioeconomic disadvantage and reduced contact with eye-care services [20, 32], so if disadvantaged individuals were less likely to attend up to amblyopia diagnosis, this could similarly have skewed our results.

It is also possible that something about the operation of the two Greater Manchester community services, reduced the perceived socioeconomic inequality. Being a community service rather than hospital-based, would reduce the distance patients must travel to an appointment for many, and there is evidence that use of NHS emergency departments reduces with distance from hospital more for disadvantaged individuals, than for advantaged individuals [33]. Therefore, it is possible that inclusion of community orthoptic services in our study reduced inequality related to distance from the clinic, compared to some other services.

For one of the two community services included in our study, patients usually see the same orthoptist. Le and Orge [9] report the importance of developing a rapport between families and clinic staff, for motivating concordance with amblyopia therapy (and improved concordance would improve therapy outcomes). It could be that the continuity of care and development of rapport is even more important for socioeconomically disadvantaged individuals, given the suggested relationship between socioeconomic disadvantage and reduced concordance [20]. If so, this service design may have reduced any socioeconomic inequality. The purpose of our study was not to compare services, and data about which orthoptist completed each visit was not recorded, so it was not possible to analyse whether this was a contributing factor. A high level of parent-reported concordance was recorded, but it is known that parents tend to over-estimate adherence with amblyopia therapy [9]. It is not known whether the accuracy of parent reports varies with SES; objective methods of measuring occlusion would be required to investigate this.

A secondary aim of this study was to determine whether there is a relationship between SES and orthoptic clinic attendance rate. No significant relationship was found, which differs from other studies [25, 32]. The differences could be related to operational aspects of services. For example, O’Colmain et al. [25] do not state whether their orthoptic clinics are community or hospital-based and as described above, inclusion of community services in our study may have reduced any socioeconomic effect. Similarly, in a study by Smith et al. [32], all centres were hospitals. This study [32] was also published ~30 years ago and socioeconomic effects are likely to have changed within that time. Furthermore, O’Colmain et al. [25] and Smith et al. [32] had much larger sample sizes of 430 and 961, respectively, versus 42 in our study. We recognise the small sample size as a limitation of our study. Compared with the more recent study by O’Colmain et al. [25], which included all vision-screening referrals, it is also possible that only including those with unilateral amblyopia, a diagnosed condition, may have increased parents’ motivation to attend appointments.

Another limitation of our study is the uneven distribution of socioeconomic scores within the sample. This is likely due to the study design, which targeted the most and least advantaged Greater Manchester boroughs, to achieve a range of scores amongst individuals. The range was achieved but numbers were limited in the middle socioeconomic groups, which will have reduced our ability to detect a relationship. This was likely enhanced by the small sample size.

Exploratory analyses of our data were performed to understand the relationship between appointment attendance rate and amblyopia therapy outcome. The analyses showed a significant (*p* = 0.007) positive relationship, so as attendance rate improved, proportion amblyopia deficit corrected also improved. This has been reported by others [25, 34], and it is expected because attendance rate could be seen as an indicator of engagement with the therapy. The relationship, coupled with our findings of no significant relationship between SES and amblyopia therapy outcome, aligns with the theory [20] that amblyopia therapy outcomes for socioeconomically disadvantaged individuals are affected by the amount of contact they have with services. In our cohort, disadvantaged individuals did not have worse outcomes, and they also attended just as well as their more advantaged peers.

It is clear, however, that attendance is not the only factor that affects therapy success because there are patients with 100% attendance who did not have 100% deficit correction in a year (Fig. 4). Results from O’Colmain et al. [25] would support this: their disadvantaged individuals, by HPI score, were more likely to have worse vision and binocularity outcomes even when they attended well.

In conclusion, a positive relationship between orthoptic clinic attendance rate and amblyopia therapy outcome is reported. In two Greater Manchester community orthoptic services, amblyopia therapy outcome, and orthoptic clinic attendance rate, were not related to individual socioeconomic scores, by postcode. Further research should relate amblyopia therapy outcomes to more individual socioeconomic factors than scores by postcode, and address the gap in data for individuals who are not engaged by current eyecare services.

## Summary

### What was known before

- Data from Scotland showed that children were 1.4 times more likely to pass pre-school vision screening if they were socioeconomically advantaged.
- For children from the same study, an association between socioeconomic disadvantage and poor attendance at eye clinic appointments was reported, and poor attendance increased the chances of residual amblyopia at discharge. In addition, children from homes needing more support from services, had worse orthoptic treatment outcomes than their more advantaged peers.
- No studies from England were found that related amblyopia treatment outcome to socioeconomic situation during childhood.

### What this study adds

- Socioeconomic disadvantage is not associated with worse clinic attendance or amblyopia treatment outcome in two Greater Manchester community orthoptic services, when measured by postcode.
- A positive relationship is found between orthoptic clinic attendance and amblyopia treatment outcome in Greater Manchester.

## Data Availability

The datasets generated during and analysed during the current study are not publicly available as the privacy of research participants could be compromised.
